# Supplementary material for: Yellow Fever Virus Interactomes Reveal Common and Divergent Strategies of Replication and Evolution for Mosquito-borne Flaviviruses
Source: bioRxiv. 2025 Jul 1:2025.06.14.659623. Preprint. [Version 2] doi: 10.1101/2025.06.14.659623 (PMC12236827; doi:10.1101/2025.06.14.659623)
Supplement: Supplement 2 [file NIHPP2025.06.14.659623v2-supplement-2.pdf]

Supplementary Files

Table S7: Antibodies

Antibody	Host Species	Dilution Used	Supplier (Catalog #)	RRID
GAPDH	Mouse	1:1000 (WB)	Fisher (PIMA515738)	AB_2537652
FLAG-M2	Mouse	1:200 (IF), 1:1000 (WB)	MilliporeSigma (F1804)	AB_262044
Strep	Mouse	1:1000 (WB), 1:500 (IF)	Qiagen (34850)	AB_2810987
HA	Mouse	1:1000 (WB), 1:200 (IF)	MilliporeSigma (H3663)	AB_262051
TOMM20	Rabbit	1:250 (IF)	ThomasScientific (11802-1-AP)	AB_2207530
Calnexin	Rabbit	1:250 (IF)	Proteintech (10427-2-AP)	AB_2069033
RBBP6	Rabbit	1:1000 (WB)	ThermoFisher (PA5-23054)	AB_11152136
YFV NS5	Rabbit	1:1000 (WB)	GeneTex (GTX134141)	AB_2887231
GRASP65	Rabbit	1:250 (IF)	From Dr. Nevan Krogan	N/A
Anti-Mouse IgG-HRP	Rabbit	1:5000 (WB)	SouthernBiotech (6170-05)	AB_2796243
Anti-Rabbit IgG-HRP	Goat	1:5000 (WB)	SouthernBiotech (4030-05)	AB_2687483
Anti-Mouse AlexaFlour-488	Goat	1:1000 (IF)	Invitrogen (A28175)	AB_2536161

bioRxiv preprint doi: https://doi.org/10.1101/2025.06.14.659623; this version posted July 1, 2025. The copyright holder for this preprint (which was not certified by peer review) is the author/funder, who has granted bioRxiv a license to display the preprint in perpetuity. It is made available under aCC-BY 4.0 International license.

Anti-Mouse AlexaFlour-555	Goat	1:1000 (IF)	Invitrogen (A21422)	AB_2535844
Anti-Rabbit AlexaFlour-488	Goat	1:1000 (IF)	Invitrogen (A11008)	AB_143165
Anti-Rabbit AlexaFlour-555	Goat	1:1000 (IF)	Invitrogen (A27039)	AB_2536100

Table S8: Oligonucleotides

Oligo Name	Sequence (5' > 3')
pcDNA / pAc5 Upstream	CGAGCTCGGATCCACTAGTC
pcDNA / pAc5 Downstream	GTTTAAACGGGCCCCTTCTC
Negative CRISPRi gRNA #1	CGCGAUAGCGCGAAUAUAUU
Negative CRISPRi gRNA #2	GCGCGAUAGCGCGAAUAUAC
ATRX CRISPRi gRNA #1	AAUUCCGAGUUUCGAGCGA
ATRX CRISPRi gRNA #2	GGUGUAGUCUUUACACGUG
DAXX CRISPRi gRNA #1	CUGGGAUGCCAUUCCACUA
DAXX CRISPRi gRNA #2	GAUCUGGAACAGAUGCAGG
DDX10 CRISPRi gRNA #1	UUCCACUUCGUCAUUGGUG
DDX10 CRISPRi gRNA #2	AGCCUUAUAUCGUCUGCAA
XRCC6 CRISPRi gRNA #1	UCCUCUGGGUACACGAACA
XRCC6 CRISPRi gRNA #2	GAGACCUCUUGGUAUCGCU
ILF2 CRISPRi gRNA #1	CUCGAACCAGCGGGCAUGU
ILF2 CRISPRi gRNA #2	CAAAUUGAAGAAGUUCGAC
RBBP6 CRISPRi gRNA #1	GGAGGGACACUAUACCCAG
RBBP6 CRISPRi gRNA #2	GUGAUCUGCAGGUCGCAGU
TMPO CRISPRi gRNA #1	CCGCCGGCACCAACAGCAA
TMPO CRISPRi gRNA #2	CUUUGCGCUGCUCCCCGGC
ZRANB2 CRISPRi gRNA #1	CCUAGACCUUGAACUUGAG
ZRANB2 CRISPRi gRNA #2	AUUAGCACUAAAUAGGCCU
STUB1 CRISPRi gRNA #1	AAUCGCGAAGAAGAAGCGC
STUB1 CRISPRi gRNA #2	GAGUGCCAGCGAAACCACG
KRR1 CRISPRi gRNA #1	AGUCAUGCUGCCUUCGAUC
KRR1 CRISPRi gRNA #2	CUGGUUCCUUCCAACCAUC
ZC3H4 CRISPRi gRNA #1	GGUGUGGUACAGCUUACAC
ZC3H4 CRISPRi gRNA #2	CCCUCCUUGUUUGAGAUCG
GNL3 CRISPRi gRNA #1	ACCUCUAGGACAACAUCGG
GNL3 CRISPRi gRNA #2	GCGCAGAGGAGGAAUUAAG
PRPF19 CRISPRi gRNA #1	CUCAUCGAGAAGUACAUUG
PRPF19 CRISPRi gRNA #2	AAUCCUGAUAGUGGCAUCG
TFB1M CRISPRi gRNA #1	GUGGAGUUGAAACACUAAA
TFB1M CRISPRi gRNA #2	CUCGAAUCGUGGGCAACGG
ZFR CRISPRi gRNA #1	AAUAGGCUACACCCGAAGC
ZFR CRISPRi gRNA #2	AUCGCCGCCGAAUGCCAGA

bioRxiv preprint doi: https://doi.org/10.1101/2025.06.14.659623; this version posted July 1, 2025. The copyright holder for this preprint (which was not certified by peer review) is the author/funder, who has granted bioRxiv a license to display the preprint in perpetuity. It is made available under aCC-BY 4.0 International license.

SARAF CRISPRi gRNA #1	GACUACAAACGCGAUCCCA
SARAF CRISPRi gRNA #2	GAUACUCAGAGUACGGUGG
EIF4ENIF1 CRISPRi gRNA #1	GAAAGAGUUGGAUACAGAC
EIF4ENIF1 CRISPRi gRNA #2	AACCACCUACUGAACCGAC
DDX54 CRISPRi gRNA #1	CGAGGAGAAGCGCGCACGG
DDX54 CRISPRi gRNA #2	UCAGAGCGGGAGCCGAAGU
CACTIN CRISPRi gRNA #1	CUGGGCGUUAUCAGCAACG
CACTIN CRISPRi gRNA #2	UUUGGCGAGAUUGAUCUUG
GAPDH qPCR Forward	ACATCGCTCAGACACCATG
GAPDH qPCR Reverse	TGTAGTTGAGGTCAATGAAGGG
RBBP6 qPCR Forward	AGATTCAAAAGGAGCGTAGGC
RBBP6 qPCR Reverse	TCGAGAATAAGAGCGTGAACG
ZRANB2 qPCR Forward	TCTTCATCACGCTCATCCTC
ZRANB2 qPCR Reverse	AGATCTCGAACGTTCTCTGG
CACTIN qPCR Forward	TACACCAACACCGACAACCC
CACTIN qPCR Reverse	TTCAGCTCCTTCTCCTCCAG
ZC3H4 qPCR Forward	CGCAAGTACAGAGAGTACAG
ZC3H4 qPCR Reverse	CCATAACTCTTGCTGTCCAT
SARAF qPCR Forward	GTTTTGGCAGTGCTTTTACA
SARAF qPCR Reverse	ACGAGTCTGAGAAGGGTGTT
TFB1M qPCR Forward	CGTAGTCGCCTCTCTGTTATGG
TFB1M qPCR Reverse	GCTGCTCTATCTTGGGCTGTATC
ZFR qPCR Forward	AGACAGAGCTTTGAAAGGAG
ZFR qPCR Reverse	CAGCAAAACAAGGTTGACAT
DAXX qPCR Forward	GTTCTGAGAATTGCGGCGAG
DAXX qPCR Reverse	GGTGGCCATAGGGGATCAAA
ATRX qPCR Forward	AGAAACAAGTTCTCCTCCAC
ATRX qPCR Reverse	CTTCCTTGCTGTTTTCCATC
GNL3 qPCR Forward	AAGGTGCTGCCAAACTGC
GNL3 qPCR Reverse	CTCCTGCTTCCTTTCTTTCC
DDX10 qPCR Forward	TTGAGGTTCTCCGAAAAGTAGG
DDX10 qPCR Reverse	ACATTTGGAGGTCGGTAGCAT
PRPF19 qPCR Forward	ATGGTTACTACCTGGCTACAGCGG
PRPF19 qPCR Reverse	TGCCTGTTGAAGCGATGAACTTGG
TMPO qPCR Forward	TGCTCGCCTCCTGCCTGTAG
TMPO qPCR Reverse	GACACAAAGCCAAGCCAGACC
EIF4ENIF1 qPCR Forward	GGTTTCACACCAGGACCACA
EIF4ENIF1 qPCR Reverse	CATCTGGGCTGTGATGGGG
XRCC6 qPCR Forward	AGTCATATTACAAAACCGAGGGC
XRCC6 qPCR Reverse	CCTTGGAGGCATCAACCAAAAA
DDX54 qPCR Forward	AGGAAGAAGAAAGGGCTCCG
DDX54 qPCR Reverse	GTCTTCCAGGTCCCAGCTTC

STUB1 qPCR Forward	AGC AGG GCA ATC GTC TGT TC
STUB1 qPCR Reverse	CAA GGC CCG GTT GGT GTA ATA
ILF2 qPCR Forward	TCTGGTTTAGTGTGAAATGGCCT
ILF2 qPCR Reverse	AGCCAGGTCCTGATTCCTCT
YFV NS5 qPCR Forward	TCCGAAGTCATGCAGCCATT
YFV NS5 qPCR Reverse	GGAAAAGAGAGAAGAAGCTGTC
